# Naples Prognostic Score: A Novel Prognostic Score in Predicting Cancer-Specific Survival in Patients With Resected Esophageal Squamous Cell Carcinoma

**DOI:** 10.3389/fonc.2021.652537

**Published:** 2021-05-28

**Authors:** Ji-Feng Feng, Jian-Ming Zhao, Sheng Chen, Qi-Xun Chen

**Affiliations:** ^1^ Department of Thoracic Oncological Surgery, Key Laboratory Diagnosis and Treatment Technology on Thoracic Oncology, Institute of Cancer Research and Basic Medical Sciences of Chinese Academy of Sciences, Cancer Hospital of University of Chinese Academy of Sciences, Zhejiang Cancer Hospital, Hangzhou, China; ^2^ Department of Thoracic Surgery, Jinhua Guangfu Hospital, Jinghua, China

**Keywords:** Naples prognostic score, esophageal squamous cell carcinoma, neutrophil to lymphocyte ratio, lymphocyte to monocyte ratio, cancer-specific survival, prognosis

## Abstract

**Background:**

Naples prognostic score (NPS) serves as a new prognostic index based on nutritional and inflammatory status in recent years. The aim of the current study was to explore the prognostic effect of NPS and to develop and validate a reliable nomogram based on NPS for individual cancer-specific survival (CSS) prediction in patients with resected ESCC without neoadjuvant therapy.

**Methods:**

The clinical data for 287 (Jan. 2010 to Jun. 2012, Training sets) and 118 (Jan. 2015 to Dec 2015, Validation sets) consecutive resected ESCC cases were retrospectively analyzed. Two NPS models based on the different cut-off values of parameters were compared. Cut-off values in model 1 were derived from previous published studies, while cut-off values in model 2 were obtained in this study based on receiver operating characteristic (ROC) curves. The relationships between NPS and clinical characteristics and CSS were analyzed. The prediction model of nomogram was developed with independent prognostic factors in the training sets and was validated in the validation sets.

**Results:**

The 5-year CSS for NPS 0, 1 and 2 were 61.9%, 34.6% and 13.4% in model 1 and 75.0%, 42.4% and 13.0% in model 2, respectively (P<0.001). Subgroup analyses revealed that NPS was also significantly associated with CSS in both model 1 and model 2 in different TNM stages. Multivariate analyses revealed that NPS was an independent prognostic marker regarding CSS in patients with resected ESCC (P<0.001). A predictive nomogram based on NPS was established and validated. The C-indexes of the nomogram in the training sets and validation sets were 0.68 and 0.72 in model 1 and 0.69 and 0.73 in model 2, respectively. These results confirmed that NPS-based nomogram was a more accurate and effective tool for predicting CSS in patients with resected ESCC.

**Conclusion:**

The current study confirmed that NPS was still a useful independent prognostic score in patients with resected ESCC. The NPS-based nomogram was successfully developed and validated, which may contribute to individual CSS prediction for resected ESCC patients.

## Introduction

Esophageal cancer (EC) is a common malignant tumor worldwide (1). EC is the 5th of incidence (18.85/100 000) and the 4th of mortality (14.11/100 000) in China (2). The two main types of EC are adenocarcinoma (AC) and squamous cell carcinoma (SCC), of which esophageal SCC (ESCC) accounts for more than 90% in China (3, 4). Although the exact reason of EC is unclear, it is thought to be the result of a combination of various factors, such as diet and lifestyle, demographic factors, environmental and genetic factors. Although the treatment is improved in recent years with the progress of science and technology, the prognosis in patients with EC is still poor. Therefore, the investigation of prognostic factors prior to treatment for patients with EC is more essential.

There is increasing evidence that inflammation and nutrition are associated with tumor prognosis. Studies published in recent years revealed that a range of inflammation-related or nutrition-related indicators, such as c-reactive protein (CRP), controlling nutritional status (CONUT), systemic inflammation score (SIS), platelet to lymphocyte ratio (PLR), neutrophil to lymphocyte ratio (NLR), prognostic nutritional index (PNI), albumin (ALB), Glasgow prognostic score (GPS) and lymphocyte to monocyte ratio (LMR), are associated with tumor prognosis (5–10). However, these inflammation-related and/or nutrition-related indicators mentioned above are to some extent deficient, and the results are still controversial. Therefore, an increasing number of comprehensive prognostic models, including inflammation-related and nutrition-related indicators, are urgently needed.

Naples Prognostic Score (NPS), a novel prognostic index combined with nutritional and inflammatory biomarkers, is recently proposed to predict the survival in patients with resected colorectal cancer (CRC) (11). The study including 562 patients with resected CRC revealed that the NPS, based on a composite score of ALB, LMR, total cholesterol (TC) and NLR, was a useful significant index for overall survival (OS) and disease-free survival (DFS). The result between NPS and OS was also confirmed in another study including 259 patients with metastatic CRC receiving first-line chemotherapy (12). According to the analyses with time-dependent receiver operating characteristic (ROC) curves, moreover, the study revealed that NPS was more sensitive than other conventional prognostic scores for OS prediction in patients with metastatic CRC. Since then, NPS has been further reported in patients with resected endometrial cancer, gastric cancer, early-stage lung cancer, osteosarcoma, and resected pancreatic cancer (13–17).

The prognostic effect of NPS in EC remains unclear. To the best of our knowledge, only one study including 165 ESCC patients with neoadjuvant therapy has concluded the associations between NPS and prognosis (18). However, the recent published study focused on patients with neoadjuvant therapy in small sample. It is well known that neoadjuvant therapy may affect the hematological indicators. Moreover, a reliable nomogram based on NPS for predicting survival in patients with resected ESCC was scarce. Therefore, the aim of this study was to determine the prognostic effect of NPS in patients with resected ESCC without neoadjuvant therapy. In addition, whether or not the NPS provides a better prognostic value than other conventional prognostic scores (GPS, CONUT, SIS and PNI) was also analyzed. Moreover, the prognostic effect of NPS in resected ESCC was verified by using a validation set. Finally, a predictive NPS-based nomogram with other clinical factors was established and validated in resected ESCC patients without neoadjuvant therapy.

## Methods

### Patient Selection

Between January 2010 and June 2012, 287 consecutive ESCC patients with radical resection in our department (Zhejiang Cancer Hospital) were retrospectively analyzed (Training set). To verify the prognostic significance of NPS and nomogram, a validation set of 118 patients with resected ESCC in our hospital from January 2015 to December 2015 was also analyzed. The present study was consistent with the declaration of Helsinki and was approved by the ethics committee of Zhejiang Cancer Hospital (No.2018-130). Patients according to the following inclusion criteria were recruited in this study: (1) patients were pathologically diagnosed with ESCC, (2) patients in stage TNM I-III with radical resection were conducted, (3) patients received no preoperative treatments, (4) patients were included without any other tumors or distant metastases, and (5) detailed clinical data were obtained within a week before surgery, including preoperative laboratory results.

### Treatment and Follow-Up

In the current study, McKeown or Ivor Lewis procedure with two-field lymphadenectomy was the main surgical resection for patients with ESCC (19, 20). McKeown and Ivor Lewis are commonly used procedures of esophagectomy for surgeons because they can make adequate lymph nodes dissection. According to the poor prognostic factors, cancer metastasis or recurrence, in the current study, the adjuvant radiotherapy (45-50.4 Gy) and/or chemotherapy (based on fluoropyrimidine and cisplatin) were conducted after operation. In our hospital, patients were generally followed up every 3 months in the first two years, every 6 months for the next three years, and once a year after five years. The follow-up results were obtained from our medical records. The last follow-up for training sets and validation sets were completed in March 2018 and April 2021, respectively.

### Data Analysis

According to the medical records, the main clinical data were collected and analyzed. The laboratory results were obtained within one week before operation, such as lymphocyte count, neutrophil count, monocytes count, ALB, CRP and TC. The NLR and LMR were defined as the ratio of neutrophil count to lymphocyte count and lymphocyte count to monocyte count, respectively. The NPS was composed of the following four serum indicators (ALB, TC, NLR and LMR) according to the previous study (11–18). ESCC has its own characteristics, and patients with ESCC are mostly malnourished, so the above hematological indicators may be different from other cancers. In order to better understand the application of NPS, therefore, two models (model 1 and model 2) were used to verify the prognostic value of NPS in resected ESCC. In model 1, the cut-off points for variables of ALB, TC, NLR and LMR were derived from previous published studies. In model 2, the cut-off values of above parameters in NPS were obtained in the current study based on ROC curves. Then the NPS was calculated into 3 groups (NPS 0, 1 and 2, respectively). The definitions of GPS, PNI, SIS and CONUT were according to the previous studies (7–10). The patients diagnosed with ESCC based on the 7th AJCC/UICC TNM staging system (21).

### Statistical Analysis

All statistical analyses in the current study were performed by using SPSS 20.0 (SPSS Inc., Chicago, IL, USA) and Medcalc 17.6 (MedCalc Software bvba, Ostend, Belgium). The chi-squared tests or Fisher’s exact test were used to evaluate the correlations grouped by NPS. The ROC curves were used to identify the sensibility and specificity of ALB, TC, NLR and LMR. The ROC curves were also performed to explore the predictive accuracy of NPS, ALB, TC, NLR and LMR. Cut-off values in model 1 for variables of ALB, TC, NLR and LMR were derived from previous published studies. In model 2, the optimal cut-off values for above variables in NPS were selected by ROC curves. The areas under the curve (AUC) for NPS (model 1 and model 2) and its components of ALB, TC, NLR and LMR, as well as other conventional prognostic scores (GPS, SIS, CONUT and PNI) were calculated and compared. The association between CSS and prognostic factors (univariate and multivariate) was analyzed by the Cox regression analyses and Kaplan-Meier methods. Hazard ratios (HRs) with 95% confidence intervals (CIs) were also calculated according to the Cox regression analyses. The predictive nomogram was established based on independent prognostic factors in the training set in multivariate analyses and was validated in the validation set by using R 3.6.0 software (22). Calibrations of the nomogram for survival prediction with 1-, 3-, and 5-year CSS were executed by comparing the training sets and validation sets. P-values less than 0.05 were considered to be statistically significant.

## Results

### Patient Characteristics

The baseline characteristics of the training sets and validation sets were shown in **Table 1**. In the training sets, there were 250 males and 37 females with the mean age of 59.0 ± 7.8 years (range: 36-78 years). There were 84 (29.3%) patients in TNM I stage, 94 (32.8%) patients in TNM II stage and 109 (37.9%) patients in TNM III stage, respectively. Adjuvant treatment was administered to 82 patients (28.6%). In the validation sets, there were 91 males and 27 females with the mean age of 60.2 ± 7.9 years (range: 41-78 years). There were more female patients in validation sets (22.9% *vs.* 12.9%, P=0.012).

**Table 1:** Baseline characteristics of ESCC patients in the training and validation sets.

	Training sets (n=287)	Validation sets (n=118)	P-value
Age (years)	59.0 ± 7.8	60.2 ± 7.9	0.179
Gender			0.012
Female	37 (12.9%)	27 (22.9%)
Male	250 (87.1%)	91 (77.1%)
Tumor length (cm)	4.2 ± 1.8	4.0 ± 1.8	0.215
Tumor location			0.634
Upper	17 (5.9%)	10 (8.4%)
Middle	132 (46.0%)	54 (45.8%)
Lower	138 (48.1%)	54 (45.8%)
Vessel invasion			0.496
Negative	246 (85.7%)	98 (83.1%)	
Positive	41 (14.3%)	20 (16.9%)	
Perineural invasion			0.197
Negative	230 (80.1%)	101 (75.3%)
Positive	57 (19.9%)	17 (24.7%)
Differentiation			0.098
Well	41 (14.3%)	19 (16.1%)
Moderate	191 (66.6%)	66 (55.9%)
Poor	55 (19.2%)	33 (28.0%)
TNM stage			0.249
I	84 (29.3%)	25 (21.2%)	
II	94 (32.8%)	43 (36.4%)
III	109 (37.9%)	50 (42.4%)
Adjuvant treatment			0.241
No	205 (71.4%)	91 (77.1%)
Yes	82 (28.6%)	27 (22.9%)
CRP (mg/L)	7.0 ± 8.2	5.8 ± 8.3	0.171
NLR	3.0 ± 1.25	3.1 ± 0.82	0.430
LMR	4.5 ± 1.74	4.0 ± 1.51	0.008
ALB (mg/dL)	4.09 ± 0.52	4.08 ± 0.71	0.824
TC (mg/dL)	179.7 ± 40.6	183.2 ± 42.9	0.435
PNI	48.9 ± 5.76	47.7 ± 7.18	0.069
GPS			0.575
0	191 (66.6%)	76 (64.4%)
1	73 (25.4%)	35 (29.7%)	
2	23 (8.0%)	7 (5.9%)	
SIS			0.020
0	96 (33.4%)	23 (19.5%)	
1	109 (38.0%)	54 (45.8%)
2	82 (28.6%)	41 (34.7%)
CONUT			0.054
0	136 (47.4%)	43 (36.4%)	
1	139 (48.4%)	65 (55.1%)
2	12 (4.2)	10 (8.5%)
NPS (model 1)			0.056
0	63 (22.0%)	14 (11.9%)
1	127 (44.3%)	56 (47.5%)	
2	97 (33.7%)	48 (40.7%)
NPS (model 2)			0.255
0	32 (11.1%)	10 (8.5%)
1	132 (46.0%)	47 (39.8%)
2	123 (42.9%)	61 (51.7%)

ESCC, esophageal squamous cell carcinoma; NPS, Naples prognostic score; CRP, C-reactive protein; NLR, neutrophil to lymphocyte ratio; LMR, lymphocyte to monocyte ratio; ALB, albumin; TC, total cholesterol; GPS, Glasgow prognostic score; TNM, tumor node metastasis; PNI, prognostic nutritional index; SIS, systemic inflammation score; CONUT, controlling nutritional status. Model 1: The cut-off values according to the previous published studies. Model 2: The cut-off values according to the ROC curves in the current study.

### Laboratory Results Analysis in the Training Sets

**Figure 1:**
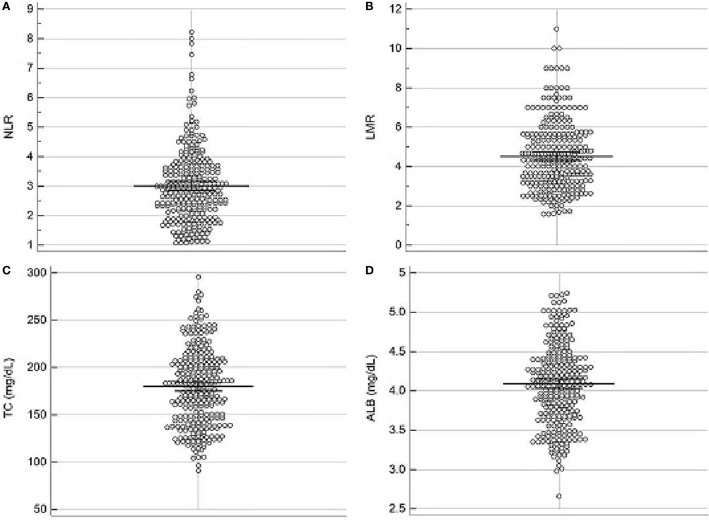
Scatter diagrams regarding NLR **(A)**, LMR **(B)**, TC **(C)** and ALB **(D)**. The mean values for NLR, LMR, ALB and TC were 3.0 ± 1.25, 4.5 ± 1.74, 4.1 ± 0.5 mg/dL and 180.0 ± 40.6 mg/dL, respectively.

**Figure 2:**
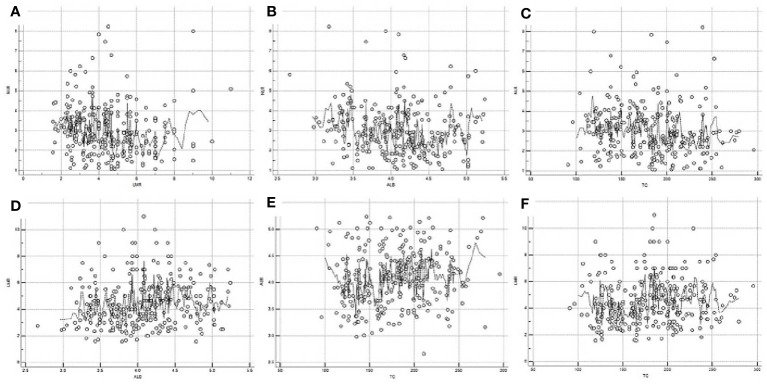
Correlation diagrams of NLR, LMR, ALB and TC. Negative correlations between NLR and LMR (r=-0.12, P=0.041, **A**), NLR and ALB (r=-0.15, P=0.011, **B**), NLR and TC (r=-0.13, P=0.026, **C**), respectively. Positive correlations between LMR and ALB (r=0.16, P=0.007, **D**), TC and ALB (r=0.12, P=0.038, **E**), respectively. No correlations between LMR and TC (r=0.12, P=0.052, **F**).

The scatter diagrams regarding NLR, LMR, ALB and TC were shown in **Figure 1**. The mean values for NLR, LMR, ALB and TC were 3.0 ± 1.25, 4.5 ± 1.74, 4.1 ± 0.5 mg/dL and 180.0 ± 40.6 mg/dL, respectively. The correlation diagrams regarding NLR, LMR, ALB and TC were shown in **Figure 2**. The results revealed that NLR was negatively correlated with LMR (r=-0.12, P=0.041), TC (r=-0.13, P=0.026) and ALB (r=-0.15, P=0.011), and the differences were statistically significant. In addition, positive correlations were found between LMR and ALB (r=0.16, P=0.007), TC and ALB (r=0.12, P=0.038), respectively. However, no correlations were found between TC and LMR (r=0.12, P=0.052).

### NPS Analysis in the Training Sets

**Figure 3:**
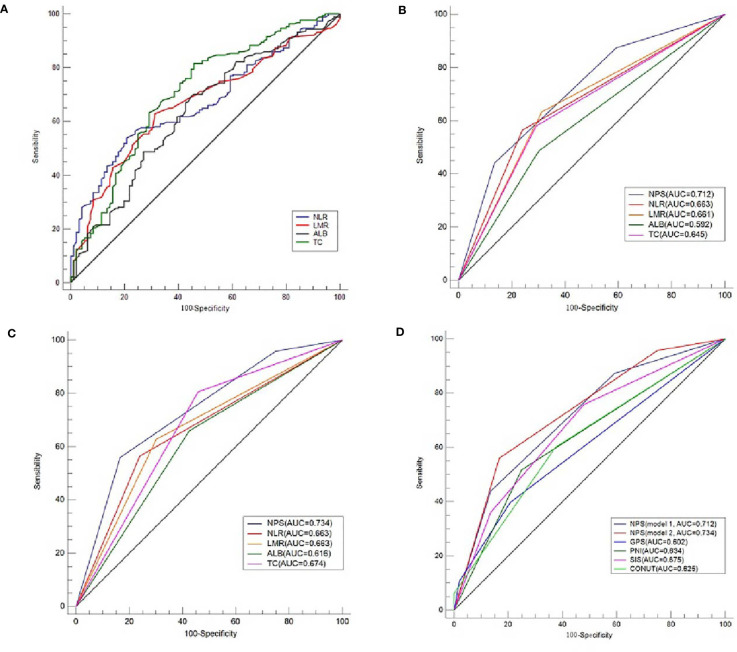
ROC analyses regarding cut-off values and AUC comparison. The optimal cut-off points for serum ALB, TC, NLR and LMR according to the ROC curves **(A)**. The optimal cut-off points for serum ALB, TC, NLR and LMR were 4.2 mg/dL, 202 mg/dL, 2.97 and 4.40, respectively. AUC comparisons between NPS and variables of ALB, TC, NLR and LMR in model 1 **(B)** and model 2 **(C)**. AUC comparisons between NPS and other conventional prognostic scores of GPS, SIS, CONUT and PNI **(D)**.

According to the previous published studies, the cut-off points for serum ALB, TC, NLR and LMR were 4.0 mg/dL, 180 mg/dL, 2.96 and 4.44, respectively. According to the ROC curves in the current study, the optimal cut-off points for serum ALB, TC, NLR and LMR were 4.2 mg/dL, 202 mg/dL, 2.97 and 4.40, respectively (**Figure 3A**). In order to better understand the application of NPS, therefore, two models (model 1 and model 2) were used to verify the prognostic value of NPS in resected ESCC. The definition of NPS based on serum ALB, NLR, TC and LMR was shown in **Table 2**. The NPS was calculated into 3 groups (NPS 0, 1 and 2, respectively). The sensibilities and specificities of serum ALB, TC, NLR and LMR were identified by ROC curves (**Table 3**). The baseline characteristics grouped by NPS in both model 1 and model 2 were shown in **Table 4**.

**Table 2:** Calculation of Naples prognostic score (NPS).

Variables	Model 1	Model 2	Points	NPS group
	Cut-off value	Cut-off value		
ALB (mg/dL)	≥ 4.0	≥ 4.2	0	NPS 1: 0 point
	< 4.0	< 4.2	1	NPS 2: 1 or 2 points
TC (mg/dL)	> 180	> 202	0	NPS 3: 3 or 4 points
	≤ 180	≤ 202	1	
NLR	≤ 2.96	≤ 2.97	0	
	> 2.96	> 2.97	1	
LMR	> 4.44	> 4.40	0	
	≤ 4.44	≤ 4.40	1	

NPS, Naples prognostic score; NLR, neutrophil to lymphocyte ratio; LMR, lymphocyte to monocyte ratio; ALB, albumin; TC, total cholesterol. Model 1: The cut-off values according to the previous published studies. Model 2: The cut-off values according to the ROC curves in the current study.

**Table 3:** Comparison of sensibility and specificity for the variables of NPS in ESCC.

	Variables	Cut-off value	Sensibility	Specificity
Model 1	ALB (mg/dL)	4.0	69.8%	49.7%
	TC (mg/dL)	180	70.8%	58.1%
	NLR	2.96	56.5%	76.0%
	LMR	4.44	67.7%	63.4%
Model 2	ALB (mg/dL)	4.2	57.3%	67.0%
	TC (mg/dL)	202	54.2%	81.7%
	NLR	2.97	56.5%	76.0%
	LMR	4.40	68.8%	62.8%

ESCC, esophageal squamous cell carcinoma; NPS, Naples prognostic score; NLR, neutrophil to lymphocyte ratio; LMR, lymphocyte to monocyte ratio; ALB, albumin; TC, total cholesterol. Model 1: The cut-off values according to the previous published studies. Model 2: The cut-off values according to the ROC curves in the current study.

**Table 4:** Comparison of AUC areas between NPS and other markers in ESCC.

		AUC	95% CI	P-value
Model 1	NPS	0.712	0.656-0.763	Reference
	NLR	0.663	0.605-0.717	0.0603
	LMR	0.661	0.603-0.715	0.0631
	ALB	0.592	0.533-0.650	0.0001
	TC	0.645	0.586-0.700	0.0077
	GPS	0.602	0.543-0.659	0.0005
	PNI	0.634	0.576-0.690	0.0038
	SIS	0.675	0.618-0.729	0.0799
	CONUT	0.625	0.567-0.682	0.0020
Model 2	NPS	0.734	0.679-0.784	Reference
	NLR	0.663	0.605-0.717	0.0055
	LMR	0.663	0.605-0.718	0.0135
	ALB	0.616	0.557-0.673	0.0001
	TC	0.674	0.616-0.728	0.0367
	GPS	0.602	0.543-0.659	0.0001
	PNI	0.634	0.576-0.690	0.0003
	SIS	0.675	0.618-0.729	0.0220
	CONUT	0.625	0.567-0.682	0.0003
Model 1 *vs.* Model 2	NPS (model 1)	0.712	0.656-0.763	Reference
	NPS (model 2)	0.734	0.679-0.784	0.1689

ESCC, esophageal squamous cell carcinoma; NPS, Naples prognostic score; CRP, C-reactive protein; NLR, neutrophil to lymphocyte ratio; LMR, lymphocyte to monocyte ratio; ALB, albumin; TC, total cholesterol; GPS, Glasgow prognostic score; TNM, tumor node metastasis; PNI, prognostic nutritional index; SIS, systemic inflammation score; CONUT, controlling nutritional status. Model 1: The cut-off values according to the previous published studies. Model 2: The cut-off values according to the ROC curves in the current study.

### ROC Analysis Regarding AUC Comparison in the Training Sets

The ROC curves regarding categorical variables for NPS and its components of ALB, TC, NLR and LMR, as well as other conventional prognostic scores (GPS, SIS, CONUT and PNI) were shown in **Figures 3B–D**. Compared with its components (ALB, TC, NLR and LMR) and other conventional prognostic scores (GPS, SIS, CONUT and PNI), NPS had the largest AUC (both in model 1 and model 2) according to the ROC curves (**Table 5**). Although the AUC of NPS in model 2 (0.734) was greater than that of NPS in model 1 (0.712), there was no statistical difference between model 1 and model 2.

**Table 5:** Comparison of baseline characteristics of ESCC patients based on NPS in training sets.

	NPS Model 1				NPS Model 2			
	0(n=97)	1(n=127)	2(n=97)	P-value	0(n=32)	1(n=132)	2(n=123)	P-value
Age (years)				0.494				0.779
≤ 60	33(52.4)	77(60.6)	59(60.8)		17(53.1)	79(59.8)	73(59.3)
> 60	30(47.6)	50(39.4)	38(39.2)		15(46.9)	53(40.2)	50(40.7)
Gender				0.078				0.193
Female	12(19.0)	18(14.2)	7(12.9)		6(18.8)	20(15.2)	11(8.9)
Male	51(81.0)	109(85.8)	90(87.1)		26(81.2)	112(84.8)	112(91.1)
Tumor length (cm)				<0.001				0.007
≤ 3.0	30(47.6)	40(31.5)	15(15.5)		14(43.8)	46(34.8)	25(20.3)
> 3.0	33(52.4)	87(68.5)	82(84.5)		18(56.2)	86(65.2)	98(79.7)
Tumor location				0.491				0.314
Upper	1(1.6)	10(7.9)	6(6.2)		0(0.0)	11(8.3)	6(4.9)
Middle	31(49.2)	59(46.5)	42(43.3)		17(53.1)	62(47.0)	53(43.1)
Lower	31(49.2)	58(45.7)	49(50.5)		15(46.9)	59(44.7)	64(52.0)
Vessel invasion				0.562				0.966
Negative	53(84.1)	112(88.2)	81(83.5)		27(84.4)	113(85.6)	106(86.2)
Positive	10(15.9)	15(11.8)	16(16.5)		5(15.6)	19(14.4)	17(13.8)
Perineural invasion				0.024				0.288
Negative	58(92.1)	99(78.0)	73(75.3)		29(90.6)	104(78.8)	97(78.9)
Positive	5(7.9)	28(22.0)	24(24.7)		3(9.4)	28(21.2)	26(21.1)
Differentiation				0.465				0.546
Well	9(14.3)	18(14.2)	14(14.4)		4(12.5)	17(12.9)	20(16.3)
Moderate	42(66.7)	90(70.9)	59(60.8)		23(71.9)	93(70.5)	75(61.0)
Poor	12(19.0)	19(14.9)	24(24.8)		5(15.6)	22(16.6)	28(22.8)
TNM stage				<0.001				0.002
I	29(46.0)	40(31.5)	15(15.5)		12(37.5)	49(37.1)	23(18.7)
II	23(36.5)	40(31.5)	31(32.0)		13(40.6)	42(31.8)	39(31.7)
III	11(17.5)	47(37.0)	51(52.5)		7(21.9)	41(31.1)	61(49.6)
Adjuvant treatment				0.921				0.408
No	46(73.0)	91(71.7)	68(70.1)		20(62.5)	98(74.2)	87(70.7)
Yes	17(27.0)	36(28.3)	29(29.9)		12(37.5)	34(25.8)	36(29.3)
CRP (mg/L)				<0.001				<0.001
≤ 10.0	58(92.1)	107(84.3)	50(51.5)		27(84.4)	115(87.1)	73(59.3)
> 10.0	5(7.9)	20(15.7)	47(48.5)		5(15.6)	17(12.9)	50(40.7)
GPS				<0.001				<0.001
0	58(92.1)	95(74.8)	38(39.2)		27(84.4)	104(78.8)	60(48.8)
1	5(7.9)	30(23.6)	38(39.2)		5(15.6)	27(20.5)	41(33.3)
2	0(0.0)	2(1.6)	21(21.6)		0(0.0)	1(0.8)	22(17.9)
PNI				<0.001				<0.001
≤ 47.5	1(1.6)	44(34.6)	78(80.4)		0(0.0)	31(23.5)	92(74.8)
> 47.5	62(98.4)	83(65.4)	19(19.6)		32(100)	101(76.5)	31(25.2)
SIS				<0.001				<0.001
0	63(100)	33(26.0)	0(0.0)		32(100)	57(43.2)	7(5.7)
1	0(0.0)	77(60.6)	32(33.0)		0(0.0)	63(47.7)	46(37.4)
2	0(0.0)	17(13.4)	65(67.0)		0(0.0)	12(9.1)	70(56.9)
CONUT				<0.001				<0.001
0	59(93.7)	60(47.2)	17(17.5)		31(96.9)	79(59.8)	26(21.1)
1	4(6.3)	67(52.8)	68(70.1)		1(3.1)	53(40.2)	85(69.1)
2	0(0.0)	0(0.0)	12(12.4)		0(0.0)	0(0.0)	12(9.8)

ESCC, esophageal squamous cell carcinoma; NPS, Naples prognostic score; CRP, C-reactive protein; GPS, Glasgow prognostic score; TNM, tumor node metastasis; PNI, prognostic nutritional index; SIS, systemic inflammation score; CONUT, controlling nutritional status. Model 1: The cut-off values according to the previous published studies. Model 2: The cut-off values according to the ROC curves in the current study.

### CSS Analysis in the Training Sets

**Figure 4:**
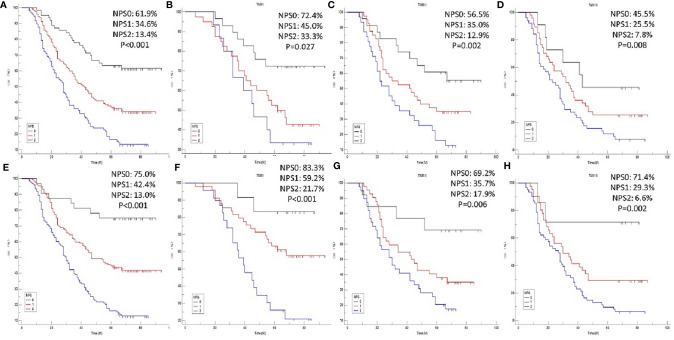
CSS analyses. Kaplan-Meier for CSS grouped by NPS in model 1 **(A)** and model 2 **(E)**. CSS analyses for NPS in subgroup analyses based on TNM stage in model 1 [TNM I: P=0.027, **(B)** TNM II: P=0.002, **(C)** TNM III: P=0.008, **(D)**] and model 2 [TNM I: P<0.001, **(F)** TNM II: P=0.006, **(G)** TNM III: P=0.002, **(H)**], respectively.

The 5-year CSS for NPS 0, 1 and 2 were 61.9%, 34.6% and 13.4% in model 1 (**Figure 4A**) and 75.0%, 42.4% and 13.0% in model 2 (**Figure 4E**), respectively (P<0.001). To better understand the prognostic significance of NPS in different TNM stages, subgroup analyses in model 1 (**Figures 4B–D**) and model 2 (**Figures 4F–H**) were also performed. In both model 1 and model 2, significant correlations between NPS and CSS were shown in different TNM stages.

### Cox Analyses With Univariate and Multivariate Analyses in the Training Sets

Univariate analyses were used to explore the significantly clinical variables associated with CSS (**Table 6**). Significant prognostic variables then were recruited in multivariate analyses. The results revealed that NPS, TNM and CRP were independent prognostic markers regarding CSS. Compared with NPS 0, patients in NPS 1 or 2 had worse 5-year CSS in model 1 (NPS 1 *vs.* NPS 0: HR=1.978, 95% CI: 1.250-3.130, P=0.004; NPS 2 *vs.* NPS 0: HR=2.903, 95% CI: 1.803-4.675, P<0.001) and model 2 (NPS 1 *vs.* NPS 0: HR=3.072, 95% CI: 1.480-6.380, P=0.003; NPS 2 *vs.* NPS 0: HR=5.239, 95% CI: 2.536-10.825, P<0.001), respectively (**Table 7**).

**Table 6:** Univariate Cox analyses of CSS in training sets.

	HR (95% CI)	P-value
Age (years)		0.925
≤ 60	1.000	
> 60	1.014 (0.760-1.353)	
Gender		0.704
Female	1.000	
Male	1.088 (0.704-1.682)	
Tumor length (cm)		0.135
≤ 3.0	1.000	
> 3.0	1.272 (0.928-1.743)	
Tumor location		0.764
Upper	1.000	
Middle	1.277 (0.664-2.456)	0.464
Lower	1.252 (0.652-2.406)	0.499
Vessel invasion		0.026
Negative	1.000	
Positive	1.540 (1.053-2.252)	
Perineural invasion		0.007
Negative	1.000	
Positive	1.585 (1.134-2.216)	
Differentiation		0.407
Well	1.000	
Moderate	1.156 (0.753-1.774)	0.508
Poor	1.394 (0.842-2.310)	0.197
TNM stage		<0.001
I	1.000	
II	1.830 (1.230-2.721)	0.003
III	2.874 (1.973-4.186)	<0.001
Adjuvant treatment		0.480
No	1.000	
Yes	1.119 (0.818-1.531)	
CRP (mg/L)		<0.001
≤ 10.0	1.000	
> 10.0	2.126 (1.559-2.901)	
NLR (model 1)		<0.001
≤ 2.96	1.000	
> 2.96	2.235 (1.676-2.982)	
NLR (model 2)		<0.001
≤ 2.97	1.000	
> 2.97	2.235 (1.676-2.982)	
LMR (model 1)		<0.001
> 4.44	1.000	
≤ 4.44	2.174 (1.616-2.923)	
LMR (model 2)		<0.001
> 4.40	1.000	
≤ 4.40	2.198 (1.636-2.953)	
ALB (mg/dL, model 1)		0.001
≥ 4.0	1.000	
< 4.0	1.615 (1.215-2.147)	
ALB (mg/dL, model 2)		<0.001
≥ 4.2	1.000	
< 4.2	1.806 (1.337-2.438)	
TC (mg/dL, model 1)		<0.001
> 180	1.000	
≤ 180	1.881 (1.409-2.511)	
TC (mg/dL, model 1)		
> 202	1.000	<0.001
≤ 202	2.586 (1.802-3.711)	
NPS (model 1)		<0.001
0	1.000	
1	2.230 (1.415-3.515)	0.001
2	4.006 (2.537-6.323)	<0.001
NPS (model 2)		<0.001
0	1.000	
1	2.937 (1.417-6.090)	0.004
2	6.313 (3.069-12.990)	<0.001
GPS		<0.001
0	1.000	
1	1.897 (1.374-2.619)	<0.001
2	3.283 (2.047-5.267)	<0.001
PNI		<0.001
≤ 47.5	1.000	
> 47.5	0.527 (0.396-0.701)	
SIS		<0.001
0	1.000	
1	1.800 (1.248-2.598)	0.002
2	2.790 (1.916-4.064)	<0.001
CONUT		<0.001
0	1.000	
1	1.474 (1.095-1.985)	0.010
2	4.127 (2.226-7.651)	<0.001

ESCC, esophageal squamous cell carcinoma; NPS, Naples prognostic score; CRP, C-reactive protein; NLR, neutrophil to lymphocyte ratio; LMR, lymphocyte to monocyte ratio; ALB, albumin; TC, total cholesterol; GPS, Glasgow prognostic score; PNI, prognostic nutritional index; SIS, systemic inflammation score; CONUT, controlling nutritional status; TNM, tumor node metastasis; CI, confidence interval; HR, hazard ratio; CSS, cancer-specific survival. Model 1: The cut-off values according to the previous published studies. Model 2: The cut-off values according to the ROC curves in the current study.

**Table 7:** Multivariate analyses regarding CSS in patients with ESCC.

		HR (95% CI)	P-value
Model 1	CRP (mg/L, > 10.0 vs. ≤ 10.0)	1.532 (1.102-2.128)	0.011
	NPS (model 1)		
	1 *vs.* 0	1.978 (1.250-3.130)	0.004
	2 *vs.* 0	2.903 (1.803-4.675)	<0.001
	TNM stage
	II *vs.* I	1.620 (1.087-2.416)	0.018
	III *vs.* I	2.196 (1.494-3.227)	<0.001
Model 2	CRP (mg/L, > 10.0 vs. ≤ 10.0)	1.618 (1.171-2.235)	0.004
	TNM stage
	II *vs.* I	1.643 (1.101-2.451)	0.015
	III *vs.* I	2.269 (1.544-3.334)	<0.001
	NPS (model 2)
	1 *vs.* 0	3.072 (1.480-6.380)	0.003
	2 *vs.* 0	5.239 (2.536-10.825)	<0.001

ESCC, esophageal squamous cell carcinoma; NPS, Naples prognostic score; CRP, C-reactive protein; TNM, tumor node metastasis; CI, confidence interval; HR, hazard ratio; CSS, cancer-specific survival. Model 1: The cut-off values according to the previous published studies. Model 2: The cut-off values according to the ROC curves in the current study.

### Nomogram Development and Validation

**Figure 5:**
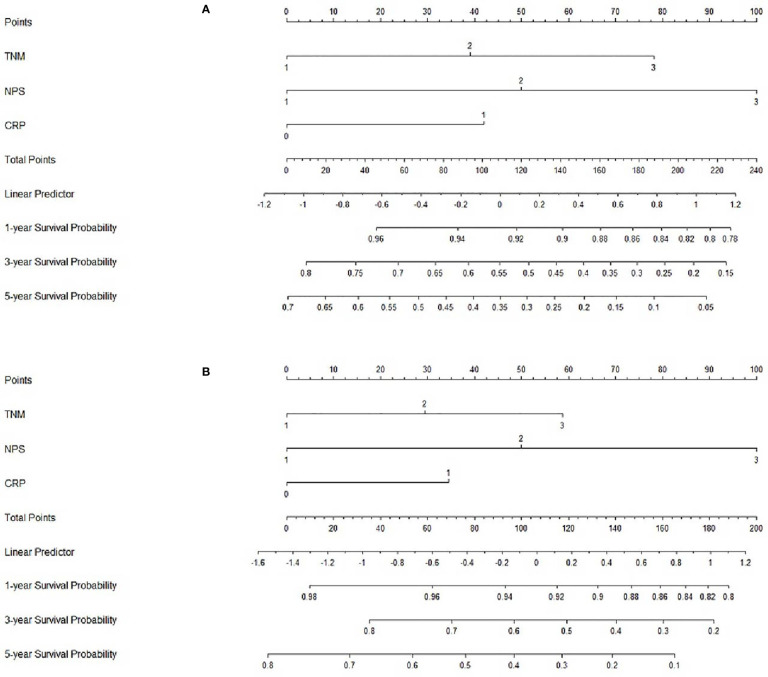
Nomogram analyses. Nomogram including NPS, TNM and CRP for predicting the 1-, 3- and 5-year CSS in patients with resected ESCC in model 1 **(A)** and model 2 **(B)**.

**Figure 6:**
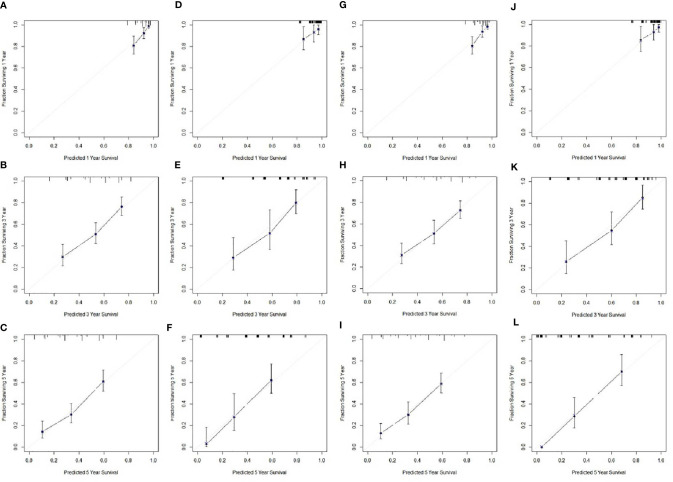
Calibration curves of the nomogram. Calibration curves presented an acceptable agreement between the training sets and validation sets. Calibration curves for CSS for 1-year, 3-year and 5-year survival nomogram calibration curves in training set **(A–C)** and validation sets **(D–F)** in model 1. Calibration curves for CSS for 1-year, 3-year and 5-year survival nomogram calibration curves in training set **(G–I)** and validation sets **(J–L)** in model 2.

Based on the analyses of prognostic factors in multivariate analyses, three variables (NPS, TNM and CRP) were selected to develop a nomogram for predicting 1-, 3- and 5-year CSS in resected ESCC patients. The predictive nomogram based on NPS in model 1 and model 2 was established in **Figure 5**. The C-indexes of the nomograms in the training sets and validation sets were 0.68 and 0.72 in model 1 and 0.69 and 0.73 in model 2, respectively. Regarding the individual 1-, 3- and 5-year CSS prediction, the calibration curves presented an acceptable agreement between the training sets and validation sets (**Figure 6**). The results in the present study confirmed the NPS-based nomogram as a more accurate and effective tool for survival prediction with 1-, 3- and 5-year CSS in patients with resected ESCC.

## Discussion

The present study confirmed the prognostic effect of the NPS, and its prognostic effect was significantly greater than other conventional prognostic scores. Compared with patients in NPS 0 group, the present study also revealed that patients in group NPS 1-2 had worse CSS. Multivariate analyses revealed that NPS, TNM stage and CRP were independent prognostic markers regarding CSS. Moreover, we firstly established and validated a new prognostic nomogram based on NPS and other independent prognostic factors. The results revealed that the NPS-based nomogram was a more accurate and effective tool for survival prediction with 1-, 3- and 5-year CSS in patients with resected ESCC.

The NPS, combined with serum TC, ALB, NLR and LMR, was initially reported by Galizia et al. in 2017 in patients undergoing surgery with CRC (11). The study including 562 patients with resected CRC revealed that the NPS was an independent significant predictor of OS and DFS. The NPS was also confirmed in another study including 259 patients with metastatic CRC receiving first-line systemic chemotherapy (12). Moreover, the results revealed that NPS was more sensitive than other conventional prognostic scores for OS prediction in patients with metastatic CRC according to the time-dependent ROC analysis. Since then, NPS has been further reported in patients with various cancers (13–17). We summarized published articles regarding the association between NPS and prognosis in cancers (**Table 8**).

**Table 8:** Comparison of NPS in published studies.

Study	Year	Country	Tumor types	Sample	Survival
Galizia	(11)	Italy	colorectal cancer	562	OS,DFS
Miyamoto	(12)	Japan	colorectal cancer	259	OS
Galizia	(16)	Italy	gastric cancer	415	OS,DFS
Li	(13)	China	endometrial cancer	1038	OS,PFS
Yang	(15)	China	osteosarcoma	133	OS,PFS
Nakagawa	(17)	Japan	pancreatic cancer	196	OS
Li	(14)	China	lung Cancer	457	OS,DFS
Kano	(18)	Japan	ESCC	165	RFS, OS
Current	2021	China	ESCC	287	CSS

ESCC, esophageal squamous cell carcinoma; NPS, Naples prognostic score; OS, overall survival; DFS, disease-free survival; PFS, progression-free survival; RFS, recurrence-free survival; CSS, cancer-specific survival.

It is well known that the components of NPS (TC, ALB, NLR and LMR) are common clinical biomarkers in daily clinical practice. The prognostic effect of NPS in EC remains unclear. Recently, Kano et al. (18) analyzed the associations between NPS and prognosis in locally advanced ESCC with neoadjuvant therapy followed by surgery. There were some differences between the Kano’s study and the current study. Firstly, the Kano’s study focused on patients with neoadjuvant therapy while the patients in the present study were recruited without any neoadjuvant therapy. Secondly, the samples in the current study were larger than that of the Kano’s study, and the results in the current study were established in the training sets and validated in the validation sets, respectively. Thirdly, the Kano’s study did not add other conventional prognostic scores in univariate and multivariate analyses. Although the NPS had the largest AUC according to ROC curves, the prognostic significance of NPS as an independent prognostic factor in univariate and multivariate analyses should be regard with caution. Fourthly, ESCC has its own characteristics, and patients with ESCC are mostly malnourished, so the above hematological indicators may be different from other cancers. In the current study, therefore, the cut-off values of parameters in NPS based on previous studies and current study were compared. Last but not least, a predictive nomogram based on NPS and other clinical factors was established and validated in the current study for survival prediction in resected ESCC patients without neoadjuvant therapy. Therefore, we performed this study to explore the prognostic effect of NPS and develop a nomogram based on NPS for survival prediction with 1-, 3- and 5-year CSS in resected ESCC patients without neoadjuvant therapy. Results from our research provided new insights regarding prognostic significance of NPS in the field of resected ESCC.

There is increasing evidence that inflammation and nutrition are associated with tumor prognosis. Studies published in recent years reported that a range of inflammation-related and/or nutrition-related indicators, such as CONUT, SIS, GPS and PNI, are associated with tumor prognosis (7–10). Compared with SIS, CONUT and PNI, NPS was considered to be the highest scoring system for grouping patients with the same OS and DFS survival rate (11). Yang et al. (15) also compared the predictive results among different independent prognostic factors through the time-dependent ROC analyses. The results demonstrated that NPS obtained the highest AUC. The above conventional prognostic scores were still controversial in patients with EC. In the current study, compared with its components (ALB, TC, NLR and LMR) and other conventional prognostic scores (GPS, SIS, CONUT and PNI), NPS had the largest AUC (both in model 1 and model 2) according to the ROC curves. GPS, SIS, CONUT and PNI were also included in our study for Cox multivariate analyses. Compared with NPS, however, the results revealed that these conventional prognostic scores were not independent significant prognostic factors.

Recently, more and more studies have revealed that nomogram based on nutritional and inflammatory status can better predict prognosis of various cancers (23–25). The nomogram can incorporate multiple factors into the prediction and consider the weight of each variable, making predictive nomogram more accurate and practical. Nomogram can also develop risk stratification and help clinicians to perform suitable treatments and survival predictions. In the current study, all three variables included in the nomogram could be obtained easily, which facilitates the application of this nomogram in clinical practice. Therefore, clinicians could use this nomogram to predict 1-, 3- and 5- year CSS rates of resected ESCC patients.

Previous published study by Galizia et al. (11) reported that NPS may have important clinical implications. They believed that improvement of inflammation and malnutrition can improve patient prognosis and prevent postoperative complications. The prognostic effect of NPS was also suitable in the present study in the clinical practice in resected ESCC patients without neoadjuvant therapy. Compared with patients in NPS 0 group, the present study revealed that patients in group NPS 1-2 had worse CSS. If patients with status of NPS 2-3, therefore, it is suggested to improve the status of inflammation and/or malnutrition before radical resection, or to conduct adjuvant therapy after surgery.

Some limitations should be mentioned in this study. Firstly, this was a retrospective study in a single-center. Secondly, the levels of serum markers such as ALB, NLR, LMR and TC may be influenced by various conditions, the applications of NPS based on these variables should be regarded with caution. Thirdly, patients treated without any preoperative therapy in the present study, which may have influenced results. Finally, the training sets and validation sets were from the same center, which may affect the generalizability of the findings in this study. Despite these limitations, the NPS-based nomogram in the present study was still an accurate and effective tool to perform survival prediction (CSS) in resected ESCC patients.

## Conclusion

In summary, the present study determined the relationships between NPS and CSS in resected ESCC patients without neoadjuvant therapy. The results indicated that NPS is still a useful prognostic score in resected ESCC patients. A new prognostic predictive model based on NPS was successfully developed and validated, which may contribute to 1-, 3- and 5-year survival prediction for resected ESCC patients.

## Data Availability Statement

The datasets presented in this study can be found in online repositories. The names of the repository/repositories and accession number(s) can be found in the article/**Supplementary Material**.

## Ethics Statement

The present study was approved by the ethics committee of Zhejiang Cancer hospital and was consistent with the declaration of Helsinki. The patients/participants provided their written informed consent to participate in this study.

## Author Contributions

J-FF, J-MZ, and Q-XC contributed to the study design. J-FF, J-MZ, SC, and Q-XC were responsible for interpretation of the results. J-MZ, CS, and J-FF contributed to statistical analysis. J-FF, J-MZ, and Q-XC were prepared for the manuscript. All authors contributed to the article and approved the submitted version.

## Funding

This study was supported by grants from Zhejiang Medical and Health Science and Technology Project (2018KY290, 2019RC129). This study was also supported by Zhejiang TCM Science and Technology Project (2021ZB034).

## Supplementary Material

The Supplementary Material for this article can be found online at: https://www.frontiersin.org/articles/10.3389/fonc.2021.652537/full#supplementary-material

## Conflict of Interest

The authors declare that the research was conducted in the absence of any commercial or financial relationships that could be construed as a potential conflict of interest.

## References

[1] Siegel RL Miller KD Jemal A. Cancer Statistics, 2015. CA: Cancer J Clin (2015) 65(1):5–29. 10.3322/caac.21254 25559415

[2] Chen W Sun K Zheng R Zeng H Zhang S Xia C. Cancer Incidence and Mortality in China, 2014. Chin J Cancer Res (2018) 30(1):1–12. 10.21147/j.issn.1000-9604.2018.01.01 29545714 PMC5842223

[3] Lin Y Totsuka Y He Y Kikuchi S Qiao Y Ueda J. Epidemiology of Esophageal Cancer in Japan and China. J Epidemiol (2013) 23(3):233–42. 10.2188/jea.JE20120162 PMC3709543 23629646

[4] Chen W Zheng R Baade PD Zhang S Zeng H Bray F. Cancer Statistics in China, 2015. CA: Cancer J Clin (2016) 66(2):115–32. 10.3322/caac.21338 26808342

[5] Feng JF Sheng C Zhao Q Chen P. Prognostic Value of Mean Platelet Volume/Platelet Count Ratio in Patients With Resectable Esophageal Squamous Cell Carcinoma: A Retrospective Study. PeerJ (2019) 7:e7246. 10.7717/peerj.7246 31328033 PMC6622162

[6] Sun Y Zhang L. The Clinical Use of Pretreatment NLR, PLR, and LMR in Patients With Esophageal Squamous Cell Carcinoma: Evidence From a Meta-Analysis. Cancer Manag Res (2018) 10:6167–79. 10.2147/CMAR.S171035 PMC6257133 30538564

[7] Castillo-Martínez L Castro-Eguiluz D Copca-Mendoza ET Pérez-Camargo DA Reyes-Torres CA Ávila EA. Nutritional Assessment Tools for the Identification of Malnutrition and Nutritional Risk Associated With Cancer Treatment. Rev Invest Clin (2018) 70(3):121–5. 10.24875/RIC.18002524 29943772

[8] Yılmaz A Tekin SB Bilici M Yılmaz H. The Significance of Controlling Nutritional Status (CONUT) Score as a Novel Prognostic Parameter in Small Cell Lung Cancer. Lung (2020) 198(4):695–704. 10.1007/s00408-020-00361-2 32424800

[9] McMillan DC. The Systemic Inflammation-Based Glasgow Prognostic Score: A Decade of Experience in Patients With Cancer. Cancer Treat Rev (2013) 39(5):534–40. 10.1016/j.ctrv.2012.08.003 22995477

[10] Hu Y Shen J Liu R Feng Z Zhang C Ling L. Prognostic Value of Pretreatment Prognostic Nutritional Index in Non-Small Cell Lung Cancer: A Systematic Review and Meta-Analysis. Int J Biol Markers (2018) 33(4):372–8. 10.1177/1724600818799876 30282502

[11] Galizia G Lieto E Auricchio A Cardella F Mabilia A Podzemny V. Naples Prognostic Score, Based on Nutritional and Inflammatory Status, is an Independent Predictor of Long-Term Outcome in Patients Undergoing Surgery for Colorectal Cancer. Dis Colon Rectum (2017) 60(12):1273–84. 10.1097/DCR.0000000000000961 29112563

[12] Miyamoto Y Hiyoshi Y Daitoku N Okadome K Sakamoto Y Yamashita K. Naples Prognostic Score is a Useful Prognostic Marker in Patients With Metastatic Colorectal Cancer. Dis Colon Rectum (2019) 62(12):1485–93. 10.1097/DCR.0000000000001484 31567920

[13] Li Q Cong R Wang Y Kong F Ma J Wu Q. Naples Prognostic Score is an Independent Prognostic Factor in Patients With Operable Endometrial Cancer: Results From a Retrospective Cohort Study. Gynecol Oncol (2020) 160(1):91–8. 10.1016/j.ygyno.2020.10.013 33081984

[14] Li S Wang H Yang Z Zhao L Lv W Du H. Naples Prognostic Score as a Novel Prognostic Prediction Tool in Video-Assisted Thoracoscopic Surgery for Early-Stage Lung Cancer: A Propensity Score Matching Study. Surg Endosc (2020). 10.1007/s00464-020-07851-7 32748268

[15] Yang Q Chen T Yao Z Zhang X. Prognostic Value of Pre-Treatment Naples Prognostic Score (NPS) in Patients With Osteosarcoma. World J Surg Oncol (2020) 18(1):24. 10.1186/s12957-020-1789-z 32000789 PMC6993441

[16] Galizia G Auricchio A de Vita F Cardella F Mabilia A Basile N. Inflammatory and Nutritional Status is a Predictor of Long-Term Outcome in Patients Undergoing Surgery for Gastric Cancer. Validation of the Naples Prognostic Score. Ann Ital Chir (2019) 90:404–16. 31814602

[17] Nakagawa N Yamada S Sonohara F Takami H Hayashi M Kanda M. Clinical Implications of Naples Prognostic Score in Patients With Resected Pancreatic Cancer. Ann Surg Oncol (2020) 27(3):887–95. 10.1245/s10434-019-08047-7 31848811

[18] Kano K Yamada T Yamamoto K Komori K Watanabe H Takahashi K. The Impact of Pretherapeutic Naples Prognostic Score on Survival in Patients With Locally Advanced Esophageal Cancer. Ann Surg Oncol (2021). 10.1245/s10434-020-09549-5 33423121

[19] Yang YS Shang QX Yuan Y Wu XY Hu WP Chen LQ. Comparison of Long-Term Quality of Life in Patients With Esophageal Cancer After Ivor-Lewis, Mckeown, or Sweet Esophagectomy. J Gastrointest Surg (2019) 23(2):225–31. 10.1007/s11605-018-3999-z 30298418

[20] Helminen O Mrena J Sihvo E. Can We Increase the Resection Rate by Minimally Invasive Approach? Experience From 100 Minimally Invasive Esophagectomies. J Oncol (2019) 2019:3809383. 10.1155/2019/3809383 30915119 PMC6409017

[21] Rice TW Rusch VW Ishwaran H Blackstone EH. Worldwide Esophageal Cancer Collaboration. Cancer of the Esophagus and Esophagogastric Junction: Data-Driven Staging for the Seventh Edition of the American Joint Committee on Cancer/International Union Against Cancer Staging Manuals. Cancer (2010) 116(16):3763–73. 10.1002/cncr.25146 20564099

[22] Iasonos A Schrag D Raj GV Panageas KS. How to Build and Interpret a Nomogram for Cancer Prognosis. J Clin Oncol (2008) 26(8):1364–70. 10.1200/JCO.2007.12.9791 18323559

[23] Li HX Chang H Xu BQ Tao YL Gao J Chen C. An Inflammatory Biomarker Based Nomogram to Predict Prognosis of Patients With Nasopharyngeal Carcinoma: An Analysis of a Prospective Study. Cancer Med (2017) 6(1):310–9. 10.1002/cam4.947 PMC5269708 27860387

[24] Zeng X Liu G Pan Y Li Y. Development and Validation of Immune Inflammation-Based Index for Predicting the Clinical Outcome in Patients With Nasopharyngeal Carcinoma. J Cell Mol Med (2020) 24(15):8326–49. 10.1111/jcmm.15097 PMC7412424 32603520

[25] Wang Y Sun K Shen J Li B Kuang M Cao Q. Novel Prognostic Nomograms Based on Inflammation-Related Markers for Patients With Hepatocellular Carcinoma Underwent Hepatectomy. Cancer Res Treat (2019) 51(4):1464–78. 10.4143/crt.2018.657 PMC6790828 30913869

